# Decision Fatigue in nurses in the COVID‐19 pandemic: A commentary

**DOI:** 10.1002/nop2.1069

**Published:** 2021-09-21

**Authors:** Zahra Hatami, Naeimeh Sarkhani, Nasrin Nikpeyma

**Affiliations:** ^1^ Department of Community Health and Geriatric Nursing School of Nursing and Midwifery Tehran University of Medical Sciences Tehran Iran; ^2^ Nursing and Midwifery Care Research Center School of Nursing and Midwifery Tehran University of Medical Sciences Tehran Iran

Dear editor

COVID‐19 is the third most prevalent disease in the 21st century and a serious threat to public health worldwide. Since the outbreak of the disease in December 2019, the responsibilities of nurses in providing health services in all roles of education, care, counseling, support, management, and leadership have increased (Dai et al., 2020). One of the most important challenges for nurses in the COVID‐19 pandemic is to work in high‐risk conditions, wear personal protective equipment, lack of sufficient and accurate information, lack of experienced nurses, unconventional work schedule, forced transfer between wards, inadequate organizational support, reduced rest time, increased working hours as well as the closure of kindergartens and problems in managing the home. The main consequence of these critical conditions has been an increase in the physical and mental workload of nurses, especially nurses working in intensive care units, which can affect clinical decision‐making and ultimately the quality of care provided to patients (Joshi, 2020). In the COVID‐19 pandemic, clinical management and decision‐making and its consequences are very important (Allan et al., 2019). Nurses face situations that can affect their decision‐making due to role strain, emotional, psychological, social, behavioral, and spiritual trauma, and post‐traumatic stress caused by the corona pandemic (McKenna, 2020). In a critical pandemic situation, due to the need for multiple decisions, access to and use of important information are difficult and nurses' cognitive function is challenged to make appropriate decisions, these conditions lead to decision bias, which is called decision fatigue (Allan et al., 2019). Decision fatigue is a phenomenon in which the store of mental resources for decision‐making is depleted and the quality of decisions made is reduced (Hirshleifer et al., 2019). Decision fatigue is the inability of a person to regulate and experience emotions (Pignatiello et al., 2020). The concept of decision fatigue is derived from the model of self‐control empowerment of Roy Baumeister et al. who have described this concept as the result of "ego depletion." They found that self‐control is typically impaired when the cognitive resources available for decision‐making are scarce. Therefore, decision fatigue temporarily reduces the quality of subsequent decisions (Bertrams et al., 2015; Sjåstad & Baumeister, 2018). Decision fatigue in nursing practice occurs when a person makes an inappropriate decision for a patient due to fatigue from numerous previous decisions and lack of response to basic needs. In a study, the prevalence of decision fatigue in nurses was reported to be 16%–36% (Vergano et al., 2020). Scott et al. reported that the prevalence of decision fatigue in nurses working in intensive care units was 29% due to physical fatigue, shift work of more than 12 hr and dissatisfaction with clinical decisions (Scott et al., 2014). Other factors associated with decision fatigue include chronic exposure to stress and complexity of work (Shirey et al., 2013), low knowledge and experience for independent decision‐making and lack of direct supervision of managers, reduced problem‐solving ability, and retention of clinical information (Oto, 2012). In addition, problems and obstacles in decision‐making such as clinical settings laws, physician and nurse participation in decision‐making, lack of support from nursing managers and lack of teamwork, high workload, and lack of time are other related factors (Mosavinasab et al., 2015). Decision fatigue has negative consequences. Allan et al. found that frequent contact with emergency nurses for health care reduced the quality of decision‐making by 5.5% and reduced the effectiveness of care due to the need for different decision‐making (Allan et al., 2019). In complex and critical situations, nurses often face doubts and uncertainties and postpone their decisions (Persson et al., 2019). Making a non‐optimal decision makes nurses feel remorse and guilt. It causes avoidant behaviors and shows passive behavior in the next decision, and this passivity can lead to simple and unenforceable decisions (Pignatiello et al., 2020). Palinkas et al. reported that decision fatigue in managers leads to the use of non‐evidence‐based methods (Palinkas et al., 2017). It seems that decision fatigue can directly affect nursing performance and patient care. An important and effective position and role of nurses in the spread of infectious diseases, such as COVID‐19, are evident. Crisis management requires proper decision‐making by the health team. The damage caused by the multistage rise of the coronavirus and its consequences has led to repeated and sometimes complex decisions made by nursing staff to deal with it, and these increasing pressures can lead to decision fatigue in nurses. Patients with COVID‐19 need high‐quality, immediate, and appropriate care, and decision fatigue can reduce the effectiveness of health care and endanger patients' health and lives. In the pandemic of COVID‐19, healthcare organizations should provide facilities for nurses, as the most important healthcare providers, so that they can make the right decision in emergency and critical situations to provide high‐quality care to patients. Excessive workload and numerous physical and mental fatigue can cause decision fatigue and its negative consequences.

## CONFLICT OF INTEREST

The authors declare that they have no competing interests.

## AUTHOR CONTRIBUTIONS

Study conception: N.N and Z.H. Data collection: N.N, Z.H and N.S. Drafting and approving of the article: N.N and N.S.

## Data Availability

The data sets used during the current study are available from the corresponding author on request.
